# Sensory augmentation for a rapid motor task in a multisensory environment

**DOI:** 10.3233/RNN-221279

**Published:** 2024-09-10

**Authors:** James Negen, Heather Slater, Marko Nardini

**Affiliations:** a School of Psychology, Liverpool John Moores University, Liverpool, UK; b Psychology Department, Durham University, Durham, UK

**Keywords:** Multisensory, visuomotor, training, augmentation, substitution

## Abstract

**Background:**

Sensory substitution and augmentation systems (SSASy) seek to either replace or enhance existing sensory skills by providing a new route to access information about the world. Tests of such systems have largely been limited to untimed, unisensory tasks.

**Objective:**

To test the use of a SSASy for rapid, ballistic motor actions in a multisensory environment.

**Methods:**

Participants played a stripped-down version of air hockey in virtual reality with motion controls (Oculus Touch). They were trained to use a simple SASSy (novel audio cue) for the puck’s location. They were tested on ability to strike an oncoming puck with the SASSy, degraded vision, or both.

**Results:**

Participants coordinated vision and the SSASy to strike the target with their hand more consistently than with the best single cue alone, *t*(13) = 9.16, *p* <.001, Cohen’s *d* = 2.448.

**Conclusions:**

People can adapt flexibly to using a SSASy in tasks that require tightly timed, precise, and rapid body movements. SSASys can augment and coordinate with existing sensorimotor skills rather than being limited to replacement use cases – in particular, there is potential scope for treating moderate vision loss. These findings point to the potential for augmenting human abilities, not only for static perceptual judgments, but in rapid and demanding perceptual-motor tasks.

Developments in sensors and wearable devices raise the question to what extent human biology can be supplemented by new devices and techniques to enhance physical and mental performance. Sensory substitution and augmentation systems (SSASy) seek to enhance human perceptual abilities by translating information about the world into a new format. For example, the EyeCane translates distance measurements to audio signals or vibrations (Maidenbaum et al., 2014). SSASys have applications to mitigating sensory deficits, such as low or absent vision. This can make them a key part of an overall strategy for (re)habilitation. With enough research, they may even become useful for everyone in terms of things like workplace safety or even augmented sport. They also provide a window into the flexibility of perception and action systems: by studying how people learn to use SSASys in different tasks and environments, we can characterize the capacity for adaptation present in human sensorimotor processing.

The present study fills a key gap by examining how a SSASy is used in a rapid motor task in a multisensory environment. SSASys have already been shown repeatedly to help people make untimed judgements in unisensory tasks (e.g., Abboud et al., 2014; Auvray et al., 2007), such as using echolocation to sense distance (Thaler & Goodale, 2016). They can also help people navigate under the right circumstances (Chebat et al., 2015; Dodsworth et al., 2020; Jicol et al., 2020; Maidenbaum et al., 2014). However, untimed judgements and navigation rely on control systems that are separate from the control of rapid movements (Goodale & Milner, 1992; Moser et al., 2008), making it unclear if such results would generalize to the present experiment. Further, some past experiments have not seen any multisensory benefit from a SASSy even when the SASSy is providing timely information that is relevant to the task (Goeke et al., 2016; König et al., 2016; Weisberg et al., 2018). In other words, while we might generally expect more sensory information to increase performance on a given task, it is not clear that this will be the case for a SASSy in a rapid motor task in a multisensory environment.

One possibility is that a SSASy can also enhance performance in a rapid motor task in a multisensory environment. This would be supported by a finding of a multisensory benefit: performance with both the SSASy and vision together would exceed performance with the best single cue. Such findings are found in many non-SSASy studies, at least for adults (Ernst & Banks, 2002; Rahnev & Denison, 2018; Rohde et al., 2016). If also found with a SSASy, this would fit with a broad view of perception and action as flexible and adaptive, in the sense of being driven by the task or computation at hand and not the specific sensory channel providing the input (Amedi et al., 2017). It would more specifically help build a case that such flexibility and adaptability are present in the systems that control rapid hand movements.

However, there are also multiple reasons why a SASSy might not enhance performance in tasks like the one here. To start, it could be that the systems that control rapid and accurate hand movement are not easily penetrable by a SSASy. Findings from interception tasks already suggest a complex and subtle interplay between vision, planning, interoception, and the muscular-skeletal system (e.g. Bootsma et al., 2016; Bootsma & van Wieringen, 1990; Goettker et al., 2019; Kirsch & Kunde, 2022; Ledouit et al., 2013), perhaps including some kinds of audio information as well (Bieńkiewicz et al., 2014; DeLucia et al., 2016). These systems may not be able to handle additional input streams. Another potential issue is that we still know relatively little about if/how a SSASy coordinates with existing perception in a multisensory environment (*though see* Goeke et al., 2016; Negen et al., 2018; Weisberg et al., 2018). It could also be that such coordination fails under time pressure – some studies of non-SSASy audio and visual cues fail to find a multimodal advantage in tasks that involve tight timing (DeLucia et al., 2016). These possibilities can only be discerned through experimentation with SSASys in rapid motor tasks in multisensory environments.

To resolve this, we asked healthy sighted participants to play a stripped-down version of air hockey in an immersive virtual environment. It involved hitting a rapidly oncoming puck before it hit the closest edge of the virtual table, similar to previous manual interception tasks (e.g. Ledouit et al., 2013). During the task, participants were presented with the visual cue of the puck moving across the table and/or a simple augmented audio cue which used pitch to signal puck left-right position on the table and timing to signal its distance (Fig. 1). During trials that included vision, vision was degraded by partially obscuring the table-top to varying degrees. We hypothesized that the presence of both cues together would allow participants to hit the puck more consistently than with either single cue alone, implying that the SSASy can penetrate rapid motor control systems and coordinate with existing sensorimotor skills.

**Fig. 1:**
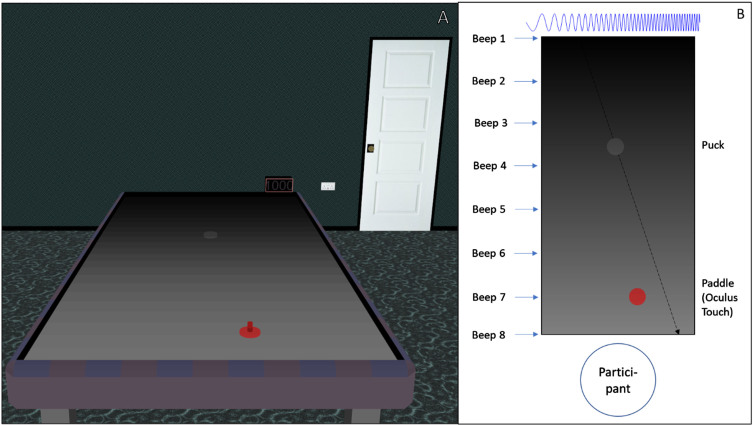
Key Methods. In immersive virtual reality, participants saw an air hockey table with a puck and a paddle. (A) A screenshot of the virtual environment. (B) Diagram here is an overhead abstraction. The goal was to hit the puck with the paddle before the puck hits the near edge. The participant moved the paddle via a motion-tracking system in their hand (Oculus Touch). A simple audio cue with lower frequency on the left and higher on the right indicated puck position when the puck crossed any of the eight beep points. Visual access was obscured by a series of black panels that had a transparency gradient. Different trial types gave the audio cue, visual access, or both.

## 1 Method

The basic task involved using a handheld motion controller to hit an oncoming virtual air hockey puck before it touched the table edge closest to the participant (Fig. 1). The study was pre-registered at https://osf.io/qws4y. All subsequent data are posted at https://osf.io/t3k9x/.

### 1.1 Participants

We recruited 20 sighted adults (9 males; age mean = 21.3 years, SD = 4.8, min = 18, max = 36) through Durham University’s Psychology Participant Pool and word of mouth. The study was approved by Durham Psychology’s Ethics Board (Reference: PSYCH-2018-12-04). They were given either £20 or 2 hours of credit towards a system allowing staff and students to participate in each other’s studies. Participants were excluded if they did not have normal vision and could not correct their vision to normal through contact lenses. The decision to test 20 adults was based on significant pilot results with 8 participants and the small telescopes approach (Simonsohn, 2015), which advocates a 2.5x increase in sample size for replication.

### 1.2 Apparatus

There were five physical devices: a laptop, Oculus Rift S (virtual reality headset), Oculus Touch (handheld motion tracking), Soundblaster SB1240 sound card, and Etymotic Research ER3SE earphones.

Worldviz Vizard 5 was used to program the virtual environment and procedure. The environment was a square room (6 m wide×6 m long×2.6 m tall) with repeating textures. In it were two familiar size references: an electrical outlet (0.146 m×0.086 m) and a door (1.981 m×0.762 m). There was also an air hockey table (1.302 m wide×2.527 m long×0.787 m tall). The surface was medium grey (RGB: 0.5, 0.5, 0.5). The puck was a cylinder (radius: 0.04 m, height: 0.02 m, RGB: 0.7, 0.7, 0.7). The paddle was a cylinder (radius: 0.04 m, height: 0.01 m, RGB: 0.8, 0.2, 0.2) with a handle in the centre that was also a cylinder (radius: 0.01 m, height: 0.04 m, RGB: 0.8, 0.2, 0.2). The paddle was constrained to stay on the table surface but otherwise followed the motion of the Oculus Touch controller in the x axis (left/right) and z axis (near/far). There was also a small box displaying a digital counter displaying the number of remaining trials. Crucially, there were also 25 black rectangles that were 0.04 m above the surface of the table and could be used to obscure/reveal the puck visually. These were tiled evenly along the length of the table. This made it impossible to see the puck when set to 100% opacity and visible throughout the trial when set to 0% opacity. The handle of the paddle was always visible.

### 1.3 Stimuli

The puck had 15 starting positions and 15 ending positions (evenly spread). It travelled down the table at a constant speed for a duration of 1.0 s. Puck position was indicated by an audio cue, visual cue, or both.

#### 1.3.1 Audio

Each auditory stimulus consisted of 8 sounds. The first began with the puck movement, the last when it touched the near edge, and the rest were spread evenly. The pitch of the sound indicated its left/right position (linear mapping from 200 Hz to 1600 Hz from left to right). Each sound consisted of 3 phases: one half-period at 60% amplitude, one full period at 100% amplitude, and the remaining 15 ms with amplitude governed by e^(-10*t*)^, where t is the proportion of this phase that has passed. PortAudio (portaudio.com) was used to minimize audio lag (approx. 2 ms).

#### 1.3.2 Visual

The visual stimulus was the virtual puck moving down the table under the obscuring rectangles. The opacity of the 25 obscuring rectangles was governed by the formula

$$\frac{1}{1+e^{-0.2(i-M)}}$$

where *i* is the index of the rectangle (0 to 24) and *M* controls the level of obscurity. This creates a gradient where the puck becomes easier to see as it approaches. After specified trigger trials in the procedure, the *M* value was varied adaptively (increased by 1 after miss; decreased by 1 after hit).

### 1.4 Procedure

#### 1.4.1 Timing

To start each trial, the rectangles above the play area were set to the desired opacities. The puck was placed at its starting location (chosen randomly). It remained there for 250 ms. Over the next 1 s, it moved down the table. If the paddle touched the puck, it was scored as a hit. The puck froze and a green line appeared over the trajectory of the puck. The game paused for 250 ms and the next trial began. If the puck was not hit before it reached the near edge, a red line appeared over its trajectory. The game paused for 1 s and then the next trial began. This means that our working definition of a “rapid” task is one in which the target movement path must be sensed, processed, planned against, and struck within 1 s to succeed. Please be aware that other definitions, such as the need for fully planned movement versus online correction, might serve to classify the present task in other ways.

#### 1.4.2 Trial Types

There were four possible trial types: audio-only, visual-only, AV, and Association. During *Audio-only* trials, the puck was not visible (100% opacity for obscuring rectangles) and the audio stimulus was presented. During *Visual-only* trials, only the visual stimulus according to the current value of *M* was presented. During *AV* trials, both were presented. For *Association* trials, the puck was fully visible (0% opacity for obscuring rectangles) and the audio stimulus was also presented.

#### 1.4.3 Session structure

The entire procedure consisted of two sessions: one training session and one testing session. Each session was on a different day, within a week of each other. Each session involved 1000 trials. The training session used a repeating pattern of the three trial types that most enable learning of the audio cue: Association, AV, and Audio-only (repeat 333x). For example, the 1st, 4th, 7th, ...  , 997th, and 1000th trials were Association. The testing session used a repeating pattern to test performance with each cue alone and the two together: AV, audio-only, visual-only, AV, and visual-only (repeat 200x). For example, the 2nd, 7th, 12th, ...  , 992nd, and 997th trials were audio-only. This means that 400 trials were AV, 400 were visual-only, and 200 were audio-only. This was done because the analysis pools all audio-only trials together but visual-only trials are separated by levels of *M* (see *Main Outcome Measures* for details) – thus the design needs more visual-only trials than audio-only trials.

#### 1.4.4 Adaptive difficulty

The procedure involved adapting the *M* value, the level of visual obscuring. An increase in *M* makes it easier to hit the puck during any trial with visual obscuring (i.e. visual-only or AV) by decreasing the opacity of the obscuring rectangles. *M* only changed between trials (never during). For the training session, *M* changed after each AV trial. A miss resulted in M increasing (i.e. easier) and a hit resulted in *M* decreasing (i.e. harder). In other words, after a miss on an AV trial, the next AV trial would be a little easier. After a hit on AV trials, the next AV trial would be a little harder. This was done simply to keep the training in a difficulty range that was challenging but not impossible as it tends towards 50% performance.

The scheme for the testing session was slightly more involved. *M* would change only after a visual-only trial (not AV). The following visual-only and AV trials would each use this updated value of *M*. Given the order, there would always be one such AV trial and one such visual-only trial after an *M* change. To be as clear as possible, here is the trial order with asterisks at the *M* changes: AV, audio-only, visual-only, *, AV, visual-only, *, ...  repeating. This has two important consequences: (1) it means that visual-only performance avoids ceiling and floor effects, instead tending towards a 50% hit rate; (2) it guarantees that each visual-only trial has a matching AV trial with the exact same value of *M* and a very similar level of training/fatigue.

#### 1.4.5 Demonstration video

A brief video showing two cycles of a testing day can be found here https://osf.io/t3k9x/files/osfstorage/63f38fa42c5c320213886e83. The reader should be warned that if this make it seem like the audio cue either lags or leads the puck, this is an artifact of encoding / replay and does not reflect the experimental experience. We have also presented just one eye since this is generally more comfortable to view outside a headset.

### 1.5 Data processing and analysis

#### 1.5.1 Exclusions

Six participants were excluded for failing to meet the criterion for learning the audio cue. The criterion was calculated with data from the second session after it was completed. For each Audio-only trial, we re-simulated the puck coming down every possible path (15 starting×15 ending = 225 paths) and re-simulated the paddle moving in the same way as during the actual trial. This was used to calculate how often the paddle movement would have hit the puck with a randomly chosen path. That was taken as a chance rate. Participants were excluded if the actual count of Audio-only hits was not significantly above the chance rate in a one-sided binomial test. (As it happens, the main result is the same without exclusions.)

#### 1.5.2 Main outcome measures

Main outcome measures were only extracted from the second session. *AV Hits* was the count of AV trials where the puck was hit. *Best Single Cue Hits* was the number of times we would expect the participant to hit the puck in AV trials if they only used their best single cue. This was calculated as

$$\sum_{M=\min\{M\}}^{\max\{M\}}\max\left\{\frac{{\mathrm{Hit}}_{\mathrm{Audio}}}{N_{\mathrm{Audio}}},\;\frac{{\mathrm{Hit}}_{\mathrm{Visual},M}}{N_{\mathrm{Visual},M}}\right\}N_{\mathrm{AV},M}$$

where *Hit* is the count of hits and *N* is the count of trials. The core idea is to go through each value of *M* and find whether performance was better with audio-only or the visual-only. The rest of the formula then multiplies and sums this to compare like-for-like with AV Hits.

#### 1.5.3 Planned analysis

We pre-registered a single one-sided paired t-test, comparing AV Hits versus Best Single Cue Hits in the direction of AV >Best Single Cue.

## 2 Results

**Fig. 2:**
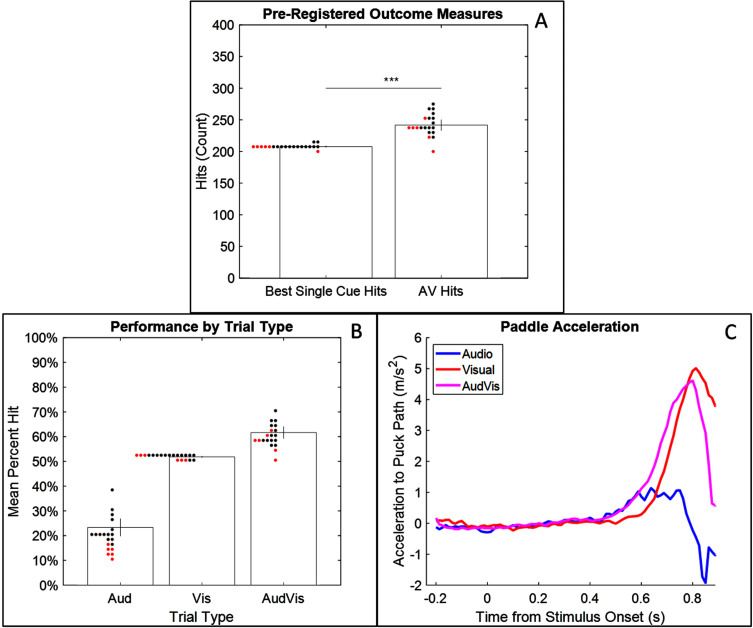
Key Results. (A) Performance with both cues was better than performance with the best single cue for 19/20 participants, p <.001. Error bars are 95% confidence intervals. Dots are individual participants. Red dots are participants who failed to show statistically significant use of the audio cue. (B) Performance by trial type. While audio-only performance was weak, it still substantially enhanced performance in combination with visual information. (C) In audio-only trials, participants accelerated the paddle towards the path of the puck early but not very sharply. In visual-only trials, they accelerated sharply but not early. When both were present, they accelerated both sharply and early.

When given both the new audio cue and the visual cue, participants hit the puck significantly more often than with the best single cue, *t*(13) = 9.16, *p* < 0.001, Cohen’s *d* = 2.448 (Fig. 2A). This confirms the main hypothesis.

### 2.1 Post-hoc exploration of acceleration

Given that participants did use both cues together to increase success, we wanted to see how this was instantiated in their movement paths. Figure 2C gives a complementary visualisation that charts the average acceleration of the paddle towards the path of the puck in different conditions. The acceleration curve for AV trials is much like the one for Visual trials except it is shifted about 100 ms earlier. This could indicate that participants were more accurate on the AV trials because they were able to begin planning and executing their strike earlier. (Acceleration here is the second derivative of distance; distance was calculated as the absolute difference on the left/right axis between the position of the paddle versus the projected position of the puck at the paddle’s point on the near/far axis.)

### 2.2 Additional checks

To be sure that participants used (and thus presumably learned) the visual cue during the training session, we checked that AV training performance was above audio-only training performance, t(19) = – 28.27, *p* < 0.001, d = 6.32. To be sure that the audio cue was useful on average overall (i.e. when including participants that were later excluded) during testing, we checked that overall audio performance was above chance using the same definition of chance as in the exclusion procedure, t(19) = 4.83, *p* < 0.001, d = 1.08. To see if the results depended on the exclusion criteria, we checked that the hit rate for both cues was above the hit rate for the best single cue overall (i.e. again including participants that were later excluded) and found the same basic pattern, t(19) = 8.59, *p* < 0.001, d = 1.922.

## 3 Discussion

The results demonstrate coordination between the SSASy (novel audio cue) and existing visual skills for a rapid motor task. *Post-hoc* acceleration analysis suggests this happens by enabling sharp, early hand movement towards the target – potentially with the SSASy providing early planning and the vision providing later refinement. This finding extends our knowledge of situations in which SSASys are useful in two ways. First, we confirmed that a SSASy can coordinate with existing sensorimotor skills in a multisensory environment (see also Negen et al., 2018; Weisberg et al., 2018). Second, we discovered that a SSASy can contribute to the kind of rapid and accurate motor control that is required for many sports and workplaces. This suggests that an appropriate SSASy can be useful in a wide variety of tasks and contexts.

This finding is important for SSASy design because it highlights additional applications. Current applications focus almost entirely on replacing vision for the blind (Thaler, 2013). While this is certainly a critical use, it may not be the only use. These results demonstrate that a SSASy can enhance sensorimotor performance by complementing existing senses (here vision) rather than replacing them. This suggests potential scope to treat moderate vision loss with sensory augmentation. There may even be potential scope to create new enhanced ways of interacting with the world for everyone – for example, playing an augmented sport or improving workplace safety. In either case, the findings here also clarify that the use of SSASys is not necessarily limited to untimed tasks or navigation; they can aid performance in a sport-like setting. These are both important areas where further research could build on these results.

Interestingly, these results demonstrate that performance with the SSASy on its own does not need to be very high for the SSASy to be helpful. While participants were able to hit approximately 50% of pucks with the visual cue in isolation, their performance with the SSASy in isolation was much worse – typically around 10–30%. Despite this, the SSASy was still able to improve performance when given in addition to vision. This suggests that by complementing existing perceptual skills, a SSASy can improve performance in multisensory contexts despite unremarkable unisensory performance. This further suggests that a SSASy should be evaluated in context of the other sensorimotor skills that a potential user possesses (rather than in isolation).

This finding is also important to theory about the organisation of perception and action because it further underlines the flexibility and adaptability of these systems (Amedi et al., 2017). Despite a lifetime of relying largely on vision to guide rapid hand movements towards targets, within two hours of training with an arbitrary new auditory pitch-position mapping, our participants effectively controlled skilled movements using this novel non-visual information. Thus, visuomotor control systems are not restricted to the solutions that worked well in the evolutionary environment (such as visual cues for intercepting rapid movement), but can adaptively integrate new information to meet the demands of the task even in a short period of time.

The finding here makes for an interesting comparison with research into sonification of the body’s movement (rather than the target’s movement). A recent review summarizes the ways that different kinds of feedback might improve motor learning (Sigrist et al., 2013). The review suggests that audio cues to the movement itself can be beneficial, though it remains unclear if this holds as task complexity increases. Further, it remains somewhat unclear if there are particular benefits from sensor-based audio cues versus verbal audio cues given by a coach. Still, both that review and the finding here point towards ability to integrate a relatively novel audio information stream into performance of a rapid motor task.

The main limitation in interpreting these results is that the SSASy was very task specific. This would not be an issue for some applications, such as the design of an augmented sport, but it would not fit every goal. It would be a different challenge to design a SASSy that would have general use. It is not obvious that such an approach would always result in the same kind of performance gains as a SSASy that is tailored to the task. With that said, the results still demonstrate that a SSASy can coordinate with vision to enhance performance on a rapid motor task. Major open questions raised by the current approach include the manner in which the visual and augmented signals interact to improve performance (e.g. integration of signals or flexible hand-over from one system to another), the levels at which neural visuomotor control pathways are influenced by the new visual signal – and how these points, and overall performance, would change with much longer training and experience.
